# Financial burden of menstrual and menopausal symptoms: productivity loss from absenteeism and presenteeism among working-age women

**DOI:** 10.1186/s12978-026-02331-y

**Published:** 2026-05-30

**Authors:** Shingo Kasahara, Rei Goto, Shu Suzuki, Haruka Sakamoto, Shohei Okamoto

**Affiliations:** 1 Graduate School of Business Administration, Keio University, 4-1-1 Hiyoshi Kohoku-ku, Yokohama Kanagawa, 223-8526 Japan; 2 Health Technology Assessment Unit, Department of Preventive Medicine and Public Health, School of Medicine, Keio University, 35 Shinanomachi, Shinjuku-ku, Tokyo, 160-8582 Japan; 3 Health and Global Policy Institute, Grand Cube 3F, Otemachi Financial City, Global Business Hub Tokyo, 1-9-2, Otemachi, Chiyoda-ku, Tokyo, 100-0004 Japan; 4 Department of Policy and Planning Sciences, University of Tsukuba, 1-1-1 Tennodai, Tsukuba, Ibaraki 305-8573 Japan; 5 Society and Science Research Unit, Tsukuba Institute for Advanced Research, University of Tsukuba, 1-1-1 Tennodai, Tsukuba, Ibaraki 305-8577 Japan; 6 Member of Health Systems Global, Ibaraki, Japan

**Keywords:** Menstruation, Menopause, Sexual and reproductive health, Financial costs, Productivity loss, Absenteeism, Presenteeism, Japan

## Abstract

**Background:**

Women’s participation in the workforce not only promotes equal opportunity but also contributes to strengthening the social and economic development by addressing declining labour force participation rates linked to population ageing. Women have unique sexual and reproductive health needs, which can hinder their workplace participation and advancement. Therefore, understanding those needs is vital to make a work environment gender-responsive and equitable to ensure that all women can fully engage in social and economic activities. This study aims to provide evidence on the financial burden of productivity loss related to menstruation and menopause.

**Methods:**

To assess the financial impact of productivity loss due to menstruation- and menopause-related symptoms, we conducted an internet-based, cross-sectional survey in Japan in September 2022. The survey included 10,000 individuals with 4,950 women aged 25–59, of which we analysed 3,046 responses from those in paid work and with necessary information. Using the validated work productivity and activity impairment questionnaire, we assessed the financial burden of productivity loss, focusing on absenteeism and presenteeism, and estimated the national-level cost among working women aged 25 to 59.

**Results:**

Results show that approximately 7.0-8.8% of working women experienced absenteeism or reduced working hours because of the symptoms. Specifically, menstruation- and menopause-related absenteeism accounted for 2–3 days off and a reduction of 2–5 working hours in the preceding three months. Additionally, 79.7% of women reported that menstrual or menopausal symptoms impacted their work productivity. Our estimate suggests that the annual financial burden of productivity loss arising from the symptoms at the national level is approximately JPY 2,296.30 billion (USD 14.65 billion), comprising JPY 323.95 billion (USD 2.07 billion) from absenteeism and JPY 1,972.35 billion (USD 12.59 billion) from presenteeism.

**Discussion:**

This study highlights the potential of addressing menstruation- and menopause-related symptoms among working-age women in generating positive economic impacts. These findings underscore the need for workplaces to implement gender-responsive strategies that meet the sexual and reproductive health needs of women in order to ensure gender equality and women’s empowerment at workplace.

## Introduction

Achieving gender equality and empowering all women and girls is a key goal of the 2030 Agenda for Sustainable Development [1]. This goal is essential for advancing health-related objectives and strengthening broader social and economic outcomes. Despite global commitments, progress remains insufficient, and women and girls continue to be left behind [2]. In particular, women’s participation in the workforce has become increasingly vital. It not only promotes equal opportunity but also addresses declining labour force participation rates linked to population ageing, especially in high-income countries. In Japan, women’s employment rates have risen, with 72.4% of women aged 15–64 employed in 2022, constituting 45.0% of the total workforce [3]. However, less than half of these women are in regular employees’ positions (i.e., a full-time, permanent positions), in contrast to over 80.0% of employed men who hold these positions. Additionally, women’s representation in senior roles remains significantly lower in Japan compared to many other countries. In 2022, women occupied only 12.9% of senior positions in Japan, while this figure typically exceeds 30.0% in other nations [3].

Health issues can impede participation in social and economic activities, including employment [4, 5]. Women face unique sexual and reproductive health needs, such as menstrual and menopausal symptoms, which can compromise their health and well-being [6, 7]. Failure to address these specific needs can hinder women’s workplace participation and advancement, leading to their underrepresentation and increased gender inequality. Additionally, this results in significant financial costs due to higher healthcare expenses and productivity losses among working-age women [8]. For instance, women with severe menstrual symptoms incur higher healthcare costs, with total expenses being 2–3 times greater for those with dysmenorrhea compared to those without [9]. The indirect costs of productivity loss also add to the financial burden, with the U.S. experiencing an estimated annual indirect cost of USD 12.0 billion [10, 11]. In Japan, the economic burden of menstrual symptoms alone is estimated at USD 8.6 billion annually (JPY 682.8 billon at the exchange rate on the publication date), with labour productivity loss accounting for 71.9% of this amount [12]. Addressing women’s health needs through a gender-responsive approach in the workplace and beyond is crucial for achieving gender equality and empowering women. This approach not only promotes gender equality but also strengthens economies by increasing labour force participation, boosting productivity, and enhancing corporate performance. Understanding the financial impacts of these health issues is essential for implementing effective gender-responsive policies and advancing gender equality and women’s empowerment.

Menstrual symptoms can lead to labour productivity losses through two primary mechanisms: absenteeism (time away from work, including tardiness and early departure) and presenteeism (reduced productivity while at work). However, evidence on the individual contributions of absenteeism and presenteeism to overall productivity loss is limited and inconsistent. Regarding absenteeism, a study of 19,254 Japanese women found that, on average, 1.0 day per year is lost due to menstrual symptoms [12]. Similarly, a study in the Netherlands reported an average of 1.3 days of absenteeism per year [13]. In contrast, presenteeism figures are notably different between studies. The Japanese study reported a 17.2% prevalence of presenteeism over the preceding three months, equating to an average of 2.4 days of productivity loss per year [12]. Meanwhile, the Dutch study recorded a higher average of 8.9 days per year [13]. These variations may be attributed to differences in study methodologies, cultural contexts, or institutional practices.

Employment status, including contract type and wage rates, can affect absenteeism and presenteeism related to perimenstrual and menopausal symptoms. For instance, regular employees may experience more presenteeism due to the opportunity costs associated with seeking care or taking time off, whereas they might also have higher rates of absenteeism due to more generous fringe benefits, such as sick leave. Despite these potential differences, to the best of our knowledge, limited evaluation has been conducted on how these factors influence productivity losses. Understanding who faces workplace challenges and their associated financial burdens is crucial for creating an environment where all women can fully engage in social and economic activities. This study aims to provide evidence on the financial burden of absenteeism and presenteeism related to menstruation and menopause, and to explore how these costs vary across different contract types.

## Methods

### Data

To assess the financial impact of productivity loss due to menstruation- and menopause-related symptoms, we conducted an internet-based, cross-sectional survey in Japan in September 2022. The survey included 10,000 individuals, with 4,950 women aged 25–59, using the Cross Marketing Inc.’s survey panel. Detailed information about the survey can be found in the previous study [14]. For this analysis, we focused exclusively on data from women respondents. The study was approved by the Ethics Review Committee of the Health Outcome Research Institute (No. 2021-03) in accordance with the Declaration of Helsinki. Informed consent to participate was obtained from all respondents before they took part in this survey.

### Evaluation of productivity loss

Using the validated work productivity and activity impairment questionnaire [15], we assessed the financial burden of productivity loss due to menstruation- and menopause-related symptoms. We modified the original scale’s timeframe from seven days to three months to better reflect the menstrual cycle and obtain more stable responses regarding productivity losses. When assessing symptoms over the past 7 days, censoring may occur if respondents did not experience symptoms because of their menstrual cycle timing, which could result in underestimation. This adjustment was also employed in a previous study [12]. We focused on the two aspects of productivity losses, namely, absenteeism and presenteeism.

#### Absenteeism

Absenteeism was estimated based on self-reported days and hours of sick leave, tardiness, and early departure from work over the preceding three months due to health issues related to menstrual symptoms (including premenstrual syndrome and dysmenorrhea) or menopausal symptoms. The financial costs of absenteeism were calculated as [Hours of absence from work due to menstrual or menopausal symptoms] × [Hourly wage].

#### Presenteeism

To evaluate presenteeism, participants rated how health problems related to menstrual or menopausal symptoms affected their work productivity over the preceding three months, using a scale from 0 (no impact) to 10 (completely prevented). The economic loss due to presenteeism per hour was calculated as [the degree of productivity reduction] × [wage per hour]. For instance, if an individual earning JPY 1,000 per hour rated their productivity impact as 6 out of 10, indicating a 40% reduction in performance, their economic loss would be JPY 400 per hour.

A previous study reported that full-time workers experienced an average of 5.20 days of decreased work efficiency due to perimenstrual and menopausal symptoms over the preceding three months, while part-time workers experienced 5.50 days [12]. Given the previous review that the duration of affective perimenstrual symptoms ranges from a few days to two weeks [16], these average values are considered conservative yet reasonable. To align the hourly loss with the absenteeism period of three months, we assumed that respondents in our survey had similar periods of reduced efficiency. Thus, we used 5.20 days for regular and contract workers, 5.50 days for part-time workers, and 5.35 days [= average (5.20, 5.50)] for self-employed individuals. The difference between full-time and part-time workers is small, but behavioural responses to symptoms may differ across employment types [17], influenced by variations in health status and access to fringe benefits such as paid sick leave. These values were adjusted to account for differences in work hours and days across individuals by applying standardised workdays based on employment type, since individuals with longer workdays per month may be affected by symptoms over a longer period compared to those with shorter workdays. This adjustment accounts for differences in work schedules within this survey and reduces the risk of using an indicator derived from a different survey that may be less relevant. The financial costs due to presenteeism over the preceding three months is formalised as follows:

$$\begin{aligned} &\left[{Financial\:costs\:due\:to\:presenteeism\:per\:3\:months}_{i}\right]\\&=\:{[Financial\:loss\:per\:hour}_{i}]\\&\times\:{[Standard\:work\:hours\:per\:day}_{i}]\\&\times\:\left[Workdays\:with\:decreased\:efficiency\:by\:employment\:type\: \right. \\& \left. \quad (i.e.,\:5.20,\:5.50\:or\:5.35\:days)\right]\\&\times\:\left[{Workdays\:in\:the\:past\:three\:months}_{i}]\right. \\& \left. \quad /[Mean\:workdays\:by\:employment\:type\right] \end{aligned}$$

where i represents each respondent in the survey. When work hours or days were missing for a respondent, we used the mean hours or days based on their employment type to calculate the financial costs.

#### Wage rates

To evaluate the wage rates and financial costs among women in paid work, we excluded individuals who were not employed, including domestic workers (*n* = 1,613) and students (*n* = 13), resulting in a size of 3,324. To gather information on respondents’ wage rates, we asked them to provide: (1) their salary system and amount (e.g., monthly, weekly, daily, hourly, or yearly wage rates) and (2) their annual pre-tax income. Annual pre-tax income was categorised into 23 ranges, from zero to JPY 20 million, with an additional “do not know” option. With their standard work hours per day and per week, we calculated respondents’ hourly wages. We primarily used the salary system and amount to estimate wage rates, but to address missing information, we also used annual pre-tax income. To address extreme values, we excluded outliers in the top and bottom 1% among women for labour hours, number of working days, and calculated hourly wages. In cases where the calculated hourly wage was significantly below the minimum wage (i.e., less than JPY 800 per hour), we recalculated the wage based on the respondent’s annual income using the median value of their income category (*n* = 335). Respondents reporting tardiness or early departure hours that exceeded their standard working hours were excluded, as these responses were considered implausible, yielding a final sample size of 3,046.

#### Relationship between wage rates and productivity losses

We conducted a linear regression analysis to descriptively visualise the relationship between wage rates and productivity losses from absenteeism and presenteeism, and to explore potential heterogeneity across employment types. The model included an interaction term between wage rates and employment type, adjusting for age, education, marital status, and the residential area and its scale. We then estimated and plotted the marginal effects of hourly wages on productivity losses.

To convert our estimates to a national level, we applied two types of weights based on the 2020 National Census: population weights and employment-type weights. Population weights were calculated based on population percentages across seven regions of Japan (1. Hokkaido and Tohoku; 2. Kita-Kanto, Koshin-etsu, and Hokuriku; 3. Minami-Kanto; 4. Tokai; 5. Kinki; 6. Chugoku and Shikoku; 7. Kyushu) and five-year age groups. Employment-type weights were derived from labour force participation rates by gender and five-year age groups for regular employees, contract workers, part-time workers, and self-employed individuals. To estimate national financial costs, we used per-capita financial costs from the survey and the number of women aged 25 to 59 by employment type from the 2020 census.

## Results

### Descriptive statistics

Table 1 summarises respondents’ characteristics by employment status, including age, education, residential area, marital status, wage rates, and absenteeism/presenteeism. The average age is 42.23 years. Regular employees have the highest proportion of university graduates (47.25%) and earn the highest average wage (JPY 2,202.32; USD 14.05), followed by self-employed (JPY 2,079.55; USD 13.27), contract workers (JPY 1,685.33; USD 10.75), and part-time workers (JPY 1,278.43; USD 8.16). Absenteeism rates range from 7.03% to 8.75%, with regular employees highest. Among those absent, average sick leave days are 2.93 (regular), 2.19 (contract), 3.33 (part-time), and 2.27 (self-employed). Reduced work hours due to health reasons are most significant among regular employees (4.93 h). Presenteeism, rated on a scale from 0 to 10, ranges from 3.55 to 4.00, with the highest value observed among regular employees.

**Table 1:** Descriptive statistics

	Regular	*n* = 1,308	Contract	*n* = 377	Part-time	*n* = 1,189	Self-employed	*n* = 172	Total	*n* = 3,046
	Mean or proportion	Standard deviation	Mean or proportion	Standard deviation	Mean or proportion	Standard deviation	Mean or proportion	Standard deviation	Mean or proportion	Standard deviation
Age	41.36	9.93	44.49	8.80	42.45	9.42	44.62	9.38	42.23	9.73
University graduate or higher	47.25%		35.19%		23.66%		35.73%		40.79%	
Hokkaido and Tohoku	8.62%		10.53%		10.79%		11.72%		9.47%	
Kita-Kanto, Koshinetsu and Hokuriku	9.99%		8.78%		12.92%		12.28%		10.44%	
Minami-Kanto	35.09%		35.92%		26.59%		26.91%		33.28%	
Tokai	10.13%		6.93%		13.76%		12.90%		10.43%	
Kinki	17.34%		17.85%		16.41%		16.66%		17.22%	
Chugoku and Shikoku	7.97%		8.54%		8.42%		6.09%		8.01%	
Kyushu	10.87%		11.45%		11.11%		13.43%		11.16%	
Unmarried	47.85%		51.21%		27.96%		35.93%		44.31%	
Married	40.19%		34.59%		63.37%		52.81%		43.98%	
Divorced/Bereaved	11.96%		14.20%		8.67%		11.25%		11.71%	
Wage rate (Japanese Yen)	2,202.32	2,173.78	1,685.33	1,681.50	1,278.43	1,269.79	2,079.55	2,232.20	1,964.51	2,016.34
Experience of absenteeism (yes/no)	8.75%		7.15%		7.03%		7.76%		8.16%	
Sick leave (days, 3 months)	0.26	1.87	0.16	0.80	0.23	1.90	0.18	0.83	0.23	1.70
Sick leave for those experienced (days, 3 months)	2.93	5.67	2.19	2.15	3.33	6.46	2.27	2.07	2.85	5.30
Reduced work hours (hours, 3 months)	0.43	2.56	0.17	1.05	0.31	2.35	0.19	1.13	0.36	2.28
Reduced work hours for those experienced (hours, 3 months)	4.93	7.29	2.38	3.24	4.38	7.84	2.39	3.47	4.35	6.84
Presenteeism (0–10)	4.00	2.72	3.55	2.74	3.66	2.70	3.91	2.70	3.87	2.73

Weighted based on population percentage by employment type and region-by-age group. The number of respondents who experienced absenteeism was 119 for regular employees, 27 for contract workers, 78 for part-time workers, and 14 for self-employed workers

## Financial costs

**Fig. 1:**
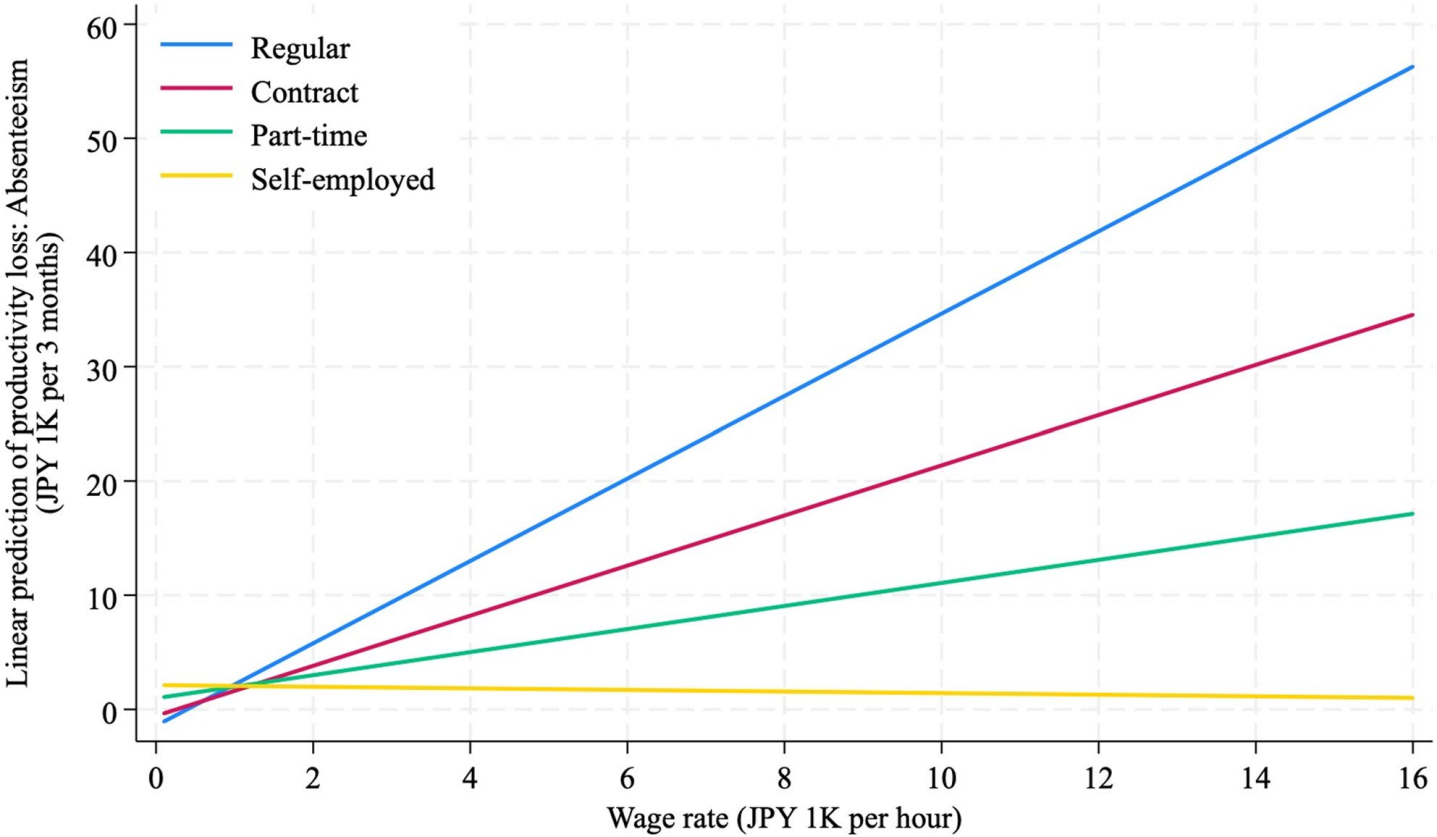
Linear prediction of productivity losses from presenteeism associated with perimenstrual and menopausal symptoms across employment types. Note: This figure illustrates the linear prediction of marginal effects of hourly wage on economic losses due to absenteeism across four employment types, based on the estimate presented in Table 2. The estimate aims to visualise the descriptive trends. The estimates are weighted by population percentage according to employment type and region-by-age group. JPY 1,000 is equivalent to USD 6.4

**Fig. 2:**
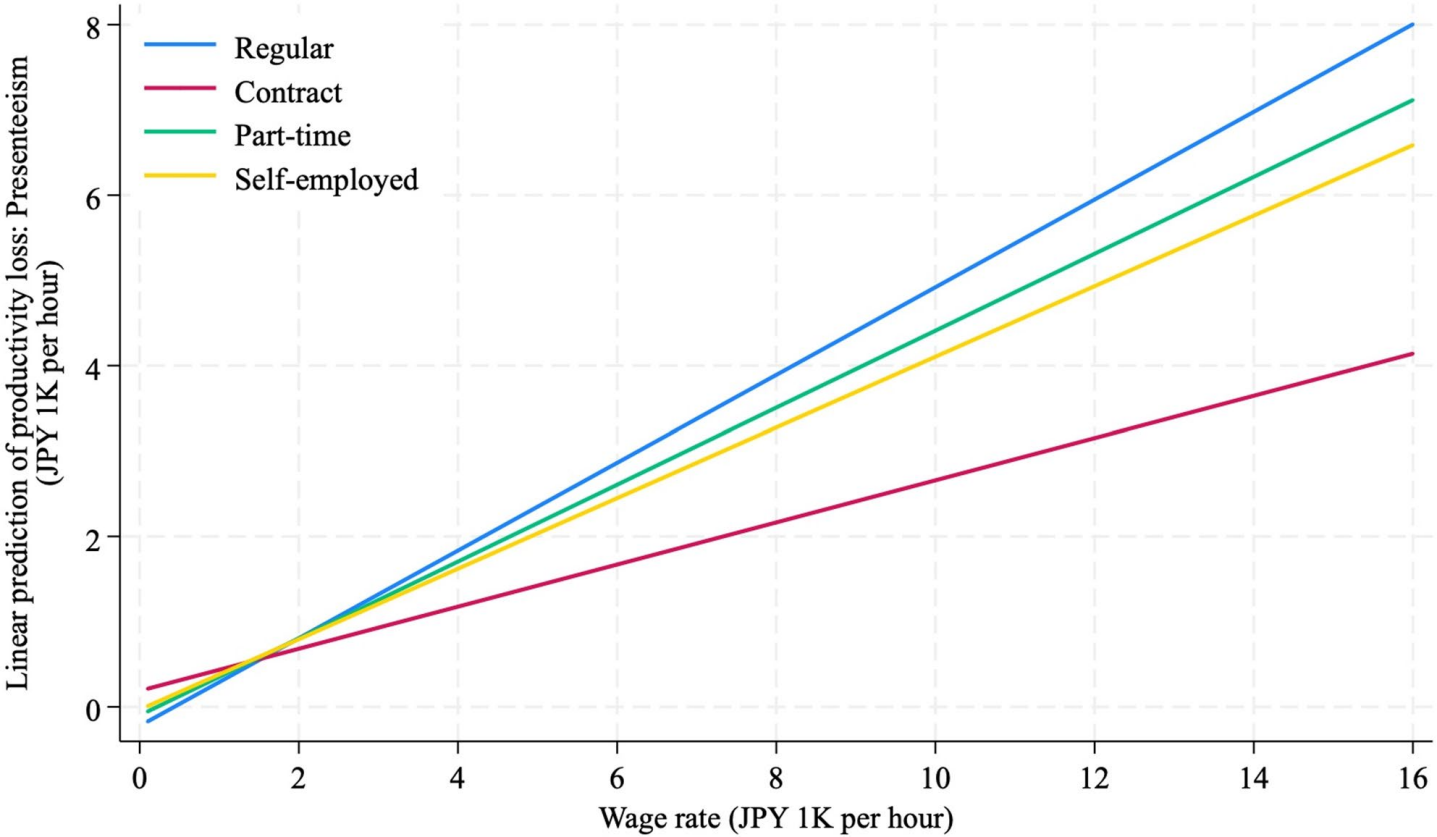
Linear prediction of productivity losses from absenteeism associated with perimenstrual and menopausal symptoms across employment types. Note: This figure illustrates the linear prediction of marginal effects of hourly wage on economic losses due to absenteeism across four employment types, based on the estimate presented in Table 4. The estimate aims to visualise the descriptive trends. The estimates are weighted by population percentage according to employment type and region-by-age group. JPY 1,000 is equivalent to USD 6.4

Figures 2 and 1, along with Tables 4 and 2, and 3, illustrate productivity losses due to perimenstrual and menopausal symptoms over the preceding three months, categorised by employment type. Table 2 presents the results of a regression analysis on the association between productivity losses and wage rates, categorised by employment type.

### Absenteeism

Figure 1 and Table 3 illustrate the financial losses associated with absenteeism, including absence, tardiness, and early departures. Over the preceding three months, 238 individuals experienced productivity losses due to these factors, with an average per-capita loss of JPY 61,330. Regular employees incurred the highest losses, averaging JPY 76,920 per capita over the same period. These financial losses were calculated based on the number of hours of tardiness, early departures, and absences, multiplied by the hourly wage. Consequently, variations in working hours and hourly wages significantly impacted the overall financial loss. The per-capita loss, calculated across the entire sample including respondents without absenteeism, was estimated at JPY 5,000 for the three-month period.

**Table 2:** Regression analysis of productivity losses and wage rates across employment types

	Absenteeism	Presenteeism
Wage rates	3.607	0.514
	(0.667, 6.547)	(0.453, 0.576)
Employment types	ref: Regular	
Contract	0.855	0.411
	(-5.093, 6.803)	(0.236, 0.587)
Part-time	2.404	0.124
	(-2.169, 6.978)	(-0.017–0.265)
Self-employed	3.551	0.190
	(-1.950, 9.051)	(0.029, 0.351)
Interaction	ref. Regular*Wage rates	
Part-time*Wage rates	-1.412	-0.267
	(-5.476, 2.651)	(-0.390, -0.145)
Contract*Wage rates	-2.597	-0.063
	(-5.772, 0.578)	(-0.171, 0.044)
Self-employed*Wage rates	-3.677	-0.101
	(-6.812, -0.541)	(-0.204, 0.003)
N	3,046	3,046

Values are coefficients with 95% confidence intervals. The models include controls for age, education (university or higher), marital status (unmarried, married, divorced/bereaved), and residential area and its scale (the same category as shown in Table 1). This estimate is conducted to visualise descriptive trends

**Table 3:** Productivity losses due to absenteeism attributable to perimenstrual and menopausal symptoms

	Only affected		All respondents	
	*N*	Loss per capita per 3 months (95% confidence interval)	*N*	Loss per capita per 3 months (95% confidence interval)
Regular	119	76.92 (44.9, 108.93)	1,308	6.73 (3.67, 9.79)
Contract	27	34.43 (17.41, 51.45)	377	2.46 (1.01, 3.92)
Part-time	78	32.88 (11.97, 53.78)	1,189	2.31 (0.84, 3.78)
Self-employed	14	17.82 (4.51, 31.13)	172	1.38 (0.20, 2.56)
Overall	238	61.33 (42.17, 80.49)	3,046	5.00 (3.37, 6.64)

Weighted based on population percentage by employment type and region-by-age group. The unit of losses is JPY 1,000, which is equivalent to USD 6.4

### Presenteeism

Figure 2 and Table 3 illustrate the financial losses due to reduced work productivity, emphasising the impact of presenteeism. Out of 3,046 women surveyed, 2,428 experienced productivity losses from presenteeism, with an average hourly per-capita loss of JPY 980. Regular employees experienced the highest hourly per-capita productivity loss, at JPY 1,130.

Assuming that the respondents experienced decreased work efficiency on certain days over the preceding three months (i.e., 5.20 days for regular and contract workers, 5.50 days for part-time workers, and 5.35 days for self-employed individuals), the total per-capita productivity loss due to presenteeism during this period would be approximately JPY 38,130. The per-capita loss, calculated across the entire sample, was estimated at JPY 30,610 for the three-month period. 

**Table 4:** Productivity losses due to presenteeism attributable to perimenstrual and menopausal symptoms

	Only affected			All respondents		
	*N*	Loss per capita per hour (95% confidence interval)	Total loss per capita per 3 months (95% confidence interval)	*N*	Loss per capita per hour (95% confidence interval)	Total loss per capita per 3 months (95% confidence interval)
Regular	1,082	1.13 (1.05, 1.22)	45.3 (41.53, 49.06)	1,308	0.92 (0.85, 1.00)	36.89 (33.66, 40.13)
Contract	295	0.73 (0.65, 0.81)	27.8 (24.10, 31.51)	377	0.56 (0.49, 0.64)	21.50 (18.39, 24.60)
Part-time	912	0.61 (0.56, 0.66)	19.04 (16.98, 21.10)	1,189	0.48 (0.44, 0.52)	14.95 (13.29, 16.61)
Self-employed	139	0.97 (0.77, 1.18)	39.17 (29.03, 49.30)	172	0.78 (0.61, 0.96)	31.52 (23.04, 40.00)
Overall	2,428	0.98 (0.93, 1.03)	38.13 (35.90, 40.37)	3,046	0.79 (0.74, 0.83)	30.61 (28.74, 32.48)

Weighted based on population percentage by employment type and region-by-age group. The unit of losses is JPY 1,000, which is equivalent to USD 6.4. To estimate the total productivity loss due to presenteeism, we assume that the respondents experienced a decrease in work efficiency for 5.20 for regular and contract workers, 5.50 for part-time workers, and 5.35 days for self-employed individuals over three months

### Financial costs at the national level

Based on the per-capita loss estimates for absenteeism and presenteeism presented in Tables 2 and 3, we calculated the national financial burden for working women aged 25 to 59 (Table 5). Overall, the annual financial costs were estimated at JPY 323.95 billion (USD 2.07 billion) for absenteeism and JPY 1,972.35 billion (USD 12.59 billion) for presenteeism. The combined total financial cost was estimated to be JPY 2,296.30 billion (USD 14.65 billion). Regular women workers incurred the highest costs, particularly from presenteeism, comprising nearly 70% of the total burden.

**Table 5:** National-level estimates of productivity losses from absenteeism and presenteeism due to perimenstrual and menopausal symptoms

	*N*	Financial burden of absenteeism per year (95% confidence interval)	Financial burden of presenteeism per year (95% confidence interval)	Financial burden of absenteeism and presenteeism per year (95% confidence interval)
Regular	9,104,246	245.09(133.65, 356.52)	1,343.42(1,225.80, 1,461.41)	1,588.51(1,359.45, 1,817.93)
Contract	738,788	7.27 (2.98, 11.58)	63.54 (54.35, 72.70)	70.81 (57.33, 84.28)
Part-time	7,073,685	65.36 (23.77, 106.95)	423.01 (376.04, 469.98)	488.37 (399.81, 576.93)
Self-employed	1,129,275	6.23 (0.90, 11.56)	142.38 (104.07, 180.68)	148.61 (104.97, 192.24)
Overall	18,045,994	323.95 (161.30, 486.61)	1,972.35 (1,760.26, 2,184.77)	2,296.30 (1,921.56, 2,671.38)

The national-level statistics were extracted from the 2020 National Census, excluding the “unknown” employment category from the estimate. The unit of losses is JPY 1 billion, which is equivalent to USD 6.4 million. The per-capita costs used to estimate the financial costs are shown in Tables 2 and 3, which show the losses per capita.

## Discussion

This study examined the financial burden of productivity loss due to menstruation- and menopause-related symptoms among working-age women across various employment types in Japan. Results show that approximately 7.0–8.8% of working women experienced absenteeism or reduced working hours because of these symptoms. Specifically, menstruation- and menopause-related absenteeism accounted for 2–3 days off and a reduction of 2–5 working hours in the preceding three months. Additionally, 79.6% of women reported that menstrual or menopausal symptoms impacted their work productivity.

Our analysis estimated that absenteeism and presenteeism resulted in average financial burdens of JPY 5,000 (USD 31.94) and JPY 30,610 per capita over three months among women survey respondents, respectively. Extrapolating this figure to a national level, the annual financial burden was estimated at JPY 323.95 billion (USD 2.07 billion) for absenteeism and JPY 1,972.35 billion (USD 12.59 billion) for presenteeism, yielding the total financial burden of JPY 2,296.30 billion (USD 14.67 billion). The productivity loss among regular employees, who typically have higher wages, was the primary contributor to the financial burden. However, significant productivity losses were also noted among other groups, including part-time workers, given their large numbers. In the absence of paid sick leave, absenteeism results in direct income loss, exacerbating the financial impact on their income. These findings highlight the unique health challenges faced by many women in the workplace and underscore the significant opportunity loss associated with neglecting the decreased productivity of women workers due to menstrual and menopausal symptoms.

Our estimates is higher than previous figures reported in Japan, which cited JPY 491.1 billion in work productivity losses, comprising JPY 283.4 billion due to absenteeism and JPY 207.7 billion due to presenteeism [12]. The discrepancy is partly due to several differences in the analyses. First, target populations are different (25–59 years old in our study versus 15–49 years old for the previous study). Second, we used more accurate wage rate measurements obtained directly from respondents, whereas the previous estimate relied on national average wages by age and employment type. Our estimate may capture heterogeneous behaviours across income groups in response to symptoms more sensitively than the previous study, including the financial burden experienced by high-income groups. Third, our study included menopausal symptoms, and 79.4% of our sample reported productivity impacts from their conditions, compared to approximately 10.7% (= 3,311 × 0.62/19,254) in previous studies, resulting in larger estimates. This discrepancy may stem from our use of a 0–10 scale to assess productivity impact, capturing even minor symptoms and thus reflecting a higher prevalence. Nevertheless, our estimate is considered to be reasonable: with an assumption that women produce 40% of the gross domestic products of Japan (JPY 600 trillion), given gender wage and employment gaps, and that they experience a 10% productivity loss for 5–6 days every three months, this would lead to the financial cost of JPY 1,920-2,300 billion. This figure is close to our estimate, suggesting that the symptoms generate the sizeable economic losses.

To address these issues, promoting societal understanding and encouraging companies to implement flexible working practices, including paid menstrual leave and workplace interventions, could be beneficial. Evidence suggests that for conditions like dysmenorrhea, physician-guided interventions are more cost-effective than self-care measures such as over-the-counter medications or acupuncture [18]. However, about 23% of women report unmet healthcare needs for menstrual and menopausal symptoms due to factors such as viewing menstrual pain as something to endure and lower health literacy [14]. Providing paid sick leave and flexible work schedules, along with employers’ responsibility to ensure contingency coverage, could mitigate substantial economic burdens by allowing women to access necessary health services and manage their symptoms effectively. Employers can also improve workers’ well-being by offering essential equipment (e.g., menstrual products, a break room, private and sanitary spaces) [19]. Equally important is increasing awareness and health literacy among all genders in the workplace so that co-workers understand these needs and help create inclusive, gender-responsive environments free from stigma, indignity, and harassment. Upstream structural interventions, including policy and legal frameworks, are also needed to comprehensively support these health needs and guarantee sexual and reproductive health rights [20].

Interpreting the findings from this study comes with caveats. First, the limitations stem from the nature of internet surveys, which can lead to non-random sampling. Although we used population and employment-type weights based on census data to address this issue, our estimates may still be biased. For instance, online surveys might over-represent individuals of higher socioeconomic status, which could lead to an overestimation of financial losses. Conversely, the estimates might be underestimated because individuals with severe conditions, who may not be included in online surveys, are underrepresented [21]. Second, this study only included individuals who were in paid work, potentially underestimating the financial losses for those who had to leave their jobs or switch to lower-paying positions due to their conditions. Third, we did not differentiate between menstrual and menopausal conditions. Future research should explore the disaggregated impacts of various health conditions on workers’ productivity and employment status to better understand and address the health needs of diverse populations. Fourth, the number of workdays with decreased efficiency from a different survey was used due to data limitations. Combined with the potential recall bias of symptoms over the past three months, better data collection methods, such as prospective studies asking respondents to record their symptoms and durations, should be pursued. Fifth, our survey was conducted in 2022 during the COVID-19 pandemic, when some individuals were allowed to work from home. This may have provided greater flexibility, potentially mitigating productivity loss. On the other hand, the fear and distress associated with COVID-19 and the infection itself could have negatively impacted menstrual and menopausal symptoms, worsening productivity loss [22, 23]. It remains inconclusive how this survey context influenced our estimates.

## Conclusions

This study highlights the substantial economic impact of menstruation- and menopause-related symptoms and the resulting productivity loss due to both absenteeism and presenteeism among working women in Japan. Specifically, absenteeism results in an estimated annual productivity loss of approximately JPY 324 billion (USD 2.1 billion), while decreased work efficiency due to these symptoms accounts for JPY 1,972 billion (USD 12.6 billion). This brings the total estimated cost to JPY 2,296 billion (USD 14.7 billion) at the national level. These findings underscore the need for workplaces to implement gender-responsive strategies that support the sexual and reproductive health needs of women to ensure gender equality and women’s empowerment at workplace. Addressing these health issues has the potential to promote gender equality but also to improving overall productivity and generating positive economic impacts for the society.

## Data Availability

Owing to ethical considerations, the data will not be made public. However, the authors will consider sharing the data upon request. Please send your request to [info@hgpi.org](mailto: info@hgpi.org) .
